# The combined effects of biotic and abiotic stress on species richness and connectance

**DOI:** 10.1371/journal.pone.0172828

**Published:** 2017-03-01

**Authors:** Devdutt Kulkarni, Frederik De Laender

**Affiliations:** Laboratory of Environmental Ecosystem Ecology, Research Unit in Environmental and Evolutionary Biology (URBE), University of Namur, Namur, Belgium; Sveriges lantbruksuniversitet, SWEDEN

## Abstract

Food web structure and species richness are both subject to biotic (e.g. predation pressure and resource limitation) and abiotic stress (e.g. environmental change). We investigated the combined effects of both types of stress on richness and connectance, and on their relationship, in a predator-prey system. To this end, we developed a mathematical two trophic level food-web model to investigate the effects of biotic and abiotic stress on food web connectance and species richness. We found negative effects of top-down and bottom-up control on prey and predator richness, respectively. Effects of top-down and bottom-up control were stronger when initial connectance was high and low, respectively. Bottom-up control could either aggravate or buffer negative effects of top-down control. Abiotic stress affecting predator richness had positive indirect effects on prey richness, but only when initial connectance was low. However, no indirect effects on predator richness were observed following direct effects on prey richness. Top-down and bottom-up control selected for weakly connected prey and highly connected predators, thereby decreasing and increasing connectance, respectively. Our simulations suggest a broad range of negative and positive richness-connectance relationships, thereby revisiting the often found negative relationship between richness and connectance in food webs. Our results suggest that (1) initial food-web connectance strongly influences the effects of biotic stress on richness and the occurrence of indirect effects on richness; and (2) the shape of the richness-connectance relationship depends on the type of biotic stress.

## Introduction

Food web structure and species richness are both subject to biotic and abiotic stress [1–2]. Biotic stress can occur through predation (top-down control on prey), resource limitation (bottom-up control on predators), or through a combination of both (mixed control [3]). When sufficiently strong, biotic stress can affect richness, e.g. when high predator richness leads to a reduction of prey diversity [4]. Species loss can lead to secondary extinctions, and thus to changes in food web structure and connectance [5–6]. Abiotic stress occurs when environmental gradients exceed tolerance limits, as such impacting food web structure and connectance [7–13]. Abiotic stressors include temperature changes and toxic chemicals, which can lead to a variety of lethal and sub-lethal effects [14]. For instance, change in temperature [15] or exposure to toxic chemicals [16] can directly affect physiological processes leading to effects on development, reproduction and survival. Just like biotic stressors, direct effects of abiotic stressors can change richness [7–11], cause secondary species loss in multi-trophic food webs [17–19] and lead to the appearance or disappearance of links between species, thereby affecting connectance [12,20]. However, despite the overwhelming evidence of biotic and abiotic stress affecting richness and connectance, the combined effects of both types of stress are far less well studied.

Since the 1970s, species richness and connectance have been shown to relate intimately. This means that food webs cannot be too complex, i.e. cannot contain many direct species interactions, and at the same time host many species [21,22]. This feature is reflected by many food webs and results in a negative relationship between connectance and species richness: a lower proportion of the potential species interactions is realised in richer food webs. An important driver of the relationship between richness and connectance, next to spatial dynamics [23], and the capacity of species to change their interactions with other species (e.g. diet shifts[24]), is the diversity of interaction types [25]. Thus, biotic stress, the term used in the present paper to denote effects of species interactions on species richness, by definition plays an important role in shaping the richness-connectance relationship. However, effects of abiotic stress on this relationship are less well studied.

The objective of the present paper is to examine the combined effects of biotic and abiotic stress on richness and connectance, and on their relationship, in a predator-prey system representing a bipartite graph. The approach we follow consists of theory development and modelling. We propose a simple theoretical framework to explore the combined effects of biotic and abiotic stress on richness and connectance in bipartite graphs making a set of well-defined assumptions. We also formalize this framework into a new model, which differs from existing food-web modelling approaches [26–28] in three important ways. First, the model is relatively parameter-sparse: 9 parameters suffice to describe how richness and connectance vary along gradients of biotic and abiotic stress, regardless of the number of species included. Second, the model for the first time unifies the effects that abiotic stress and biotic stress have on species-level fitness, as suggested elsewhere [29–31], by using the same mathematical formulation for both types of stress. Third, the model combines stochasticity (prevailing in the absence of stress) with species selection (prevailing at high biotic and/or abiotic stress), acknowledging the importance of both stochastic and deterministic drivers of food web assembly [32]. We present simulations with our model, representing a full factorial design of the biotic stressor type (three levels: top-down; bottom-up; mixed control), abiotic stressor type (two levels: affecting predator reproduction; affecting prey reproduction), and initial connectance (two levels: low and high). Under top-down control, predator abundance affected prey survival. Under bottom-up control, prey abundance affected predator reproduction. Under mixed control both effects occurred. Using this model, we first ask whether abiotic stress can indirectly alter diversity by changing biotic stress levels, i.e. if predator (prey) richness decreases (increases) following abiotic stress on prey and predators. Second, we evaluate the corresponding changes in connectance and the consequences for the richness-connectance relationship.

## Materials and methods

### Theory and model description

The theory we present combines stochastic processes with the effects of abiotic and biotic stressors on demographic rates, following a recently developed approach [9]. The stochastic processes are based on the neutral theory of biodiversity [33] and assume that individuals have identical individual-level traits, and that changes in relative abundance in a local community (the case we consider here) only occur through random death, reproduction, and dispersal. A version of the neutral model only including local processes has been previously modified to include abiotic stress taking into account both intra- and interspecific tolerance variability [9]. In the present paper, we extended this model to include (1) two communities occupying distinct trophic levels (called ‘prey’ and ‘predators’) (2) effects of abiotic stress on prey and predator survival and reproduction and (3) effects of predation and resource limitation (biotic stress) on prey survival and predator reproduction, respectively. Immigration occurs between a spatially non-explicit mainland (‘meta-community’) and the two considered local communities. Its rate depends on an immigration probability *m* and species-specific relative abundances in the mainland. Abiotic stress is assumed to not affect the mainland [9].

#### Death

A species *j* can lose an individual through death in two non-mutually exclusive ways: by chance and by biotic stress (predation). Effects of abiotic stress on death rates can be easily considered, but are left out for simplicity. Death by chance for species *j* (or ‘background stochastic death’) is simply N_j_/N, where N_j_ is the abundance of species *j* and N = ∑ N_j_ is the total number of individuals within the considered community.

Death by predation can occur when the corresponding biotic stress levels exceed the corresponding individual-level thresholds. The probability to die by predation is calculated by dividing the number of individuals from species *j* susceptible to death by predation by all individuals in the community (i.e. all species) that are susceptible to death by predation. In order to be susceptible, c_p_ (the critical individual-level threshold for death by predation) should be exceeded by the total predator abundance *pred*. This leads to the following equation:

P(death by chance or predation for species *j*)

$$=\frac{\text{P}\left[{\left(\text{c}>{\text{c}}_{\text{m}}\right)}_{\text{j}}\cup {\left(\text{pred}>{\text{c}}_{\text{p}}\right)}_{\text{j}}\right]\cdot {\text{N}}_{\text{j}}}{\sum_{\text{i}=1}^{\text{n}}\left[\text{P}\left[{\left(\text{c}>{\text{c}}_{\text{m}}\right)}_{\text{i}}\cup {\left(\text{pred}>{\text{c}}_{\text{p}}\right)}_{\text{i}}\right]\cdot {\text{N}}_{\text{i}}\right]}+\frac{{\text{N}}_{\text{j}}}{\sum_{\text{i}=1}^{\text{n}}{\text{N}}_{\text{i}}}-\frac{\text{P}\left[{\left(\text{c}>{\text{c}}_{\text{m}}\right)}_{\text{j}}\cup {\left(\text{pred}>{\text{c}}_{\text{p}}\right)}_{\text{j}}\right]\cdot {\text{N}}_{\text{j}}}{\sum_{\text{i}=1}^{\text{n}}\left[\text{P}\left[{\left(\text{c}>{\text{c}}_{\text{m}}\right)}_{\text{i}}\cup {\left(\text{pred}>{\text{c}}_{\text{p}}\right)}_{\text{i}}\right]\cdot {\text{N}}_{\text{i}}\right]}\cdot \frac{{\text{N}}_{\text{j}}}{\sum_{\text{i}=1}^{\text{n}}{\text{N}}_{\text{i}}}$$ (1)

where:

$$\text{P}{\left(\text{pred}>{\text{c}}_{\text{p}}\right)}_{\text{i}}=1-\frac{1}{1+{\left(\frac{\text{pred}}{{\text{c}}_{\text{p},50,\text{i}}}\right)}^{s_{pi}}}$$ (2)

c_m,50,i_ and c_p,50,i_ are the species-mean threshold for species *j*; *s*_*pi*_ is the slope, representing intraspecific tolerance variability; c_m_ is the critical individual-level threshold for death by abiotic stress. Because c_p,50,i_ is species-mean tolerance, it is not an individual-level parameter. Eq 2 quantifies how individuals within a species differ in sensitivity to predation. The slope represents the steepness of this distribution and, therefore, intraspecific tolerance variability [9].

The value of ‘pred’ depends on food web topology, which can be formalised using a food web (or ‘adjacency’) matrix **f**. This is a (q × q) matrix (q species in total, including all prey and predator species). The first k rows and columns represent the k prey species; the remaining q-k rows and columns represent predators. For every predator *j* eating a prey *i*, a ‘1’ is placed at the corresponding element **f**[i,j]. If organised in this way, ‘pred’ for species *j* is the *j*^th^ element of the matrix product **f** × **n**, where **n** is the (q × 1) vector of all q species abundances. For a top predator, *pred* will always equal zero so that Eq 1 simply collapses to background mortality (the second term in Eq 1).

#### Reproduction

A species *j* can gain an individual through immigration (see first paragraph of ‘Material and methods‘) or through reproduction. The probability that an individual from species *j* reproduces is given by dividing the number of individuals from species *j* eligible for reproduction by all individuals in the community (i.e. from all species) that are eligible for reproduction. An individual is eligible for reproduction when biotic (resource limitation) and abiotic stress do not impede this. Biotic stress (resource limitation) occurs when the total amount of resource, i.e. the summed densities of all prey available to individual of species *j*, is lower than c_f_ (critical threshold for reproduction impairment by resource limitation). Abiotic stress occurs when the abiotic stress level c exceeds c_r_ (critical threshold for reproduction impairment by abiotic stress). This leads to:

P(an individual from species *j* reproduces)

$$=\frac{\text{P}\left({\left(\text{resource}>{\text{c}}_{\text{f}}\right)}_{\text{j}}\cap {\left(\text{c}<{\text{c}}_{\text{r}}\right)}_{\text{j}}\right)\cdot {\text{N}}_{\text{j}}}{\sum_{\text{i}=1}^{\text{n}}\left[\text{P}\left({\left(\text{resource}>{\text{c}}_{\text{f}}\right)}_{\text{i}}\cap {\left(\text{c}<{\text{c}}_{\text{r}}\right)}_{\text{i}}\right)\cdot {\text{N}}_{\text{i}}\right]}$$ (3)

where:

$$\text{P}{\left(\text{resource}>{\text{c}}_{\text{f}}\right)}_{\text{i}}=1-\frac{1}{1+{\left(\frac{\text{resource}}{{\text{c}}_{\text{f},50,\text{i}}}\right)}^{{\text{s}}_{fi}}}$$ (4)

$$\text{P}{\left(\text{c}<{\text{c}}_{\text{r}}\right)}_{\text{i}}=\frac{1}{1+{\left(\frac{\text{c}}{{\text{c}}_{\text{r},50,\text{i}}}\right)}^{{\text{s}}_{ri}}}$$ (5)

Again, c_f,50,i_ and c_r,50,i_ are the species-mean thresholds; *s*_*ri*_ is the slope, representing intraspecific variability. Because c_f_ and c_r_ are species-mean tolerances, they are not individual-level parameters. Eqs 4 and 5 quantify how individuals within a species differ in sensitivity to resource limitation and abiotic stress respectively. The slope represents the steepness of this distribution and therefore intraspecific tolerance variability [9]. *Resource* is simply the *j*^th^ element of the matrix product **f**^**T**^ x **n**, with **f** and **n** as in section ‘Death’. For species that suffer no resource limitation, this equation simplifies to:

P (an individual from species *j* reproduces)

$$=\frac{\text{P}{\left(\text{c}<{\text{c}}_{\text{r}}\right)}_{\text{j}}\cdot {\text{N}}_{\text{j}}}{\sum_{\text{i}=1}^{\text{n}}\left[\text{P}{\left(\text{c}<{\text{c}}_{\text{r}}\right)}_{\text{i}}\cdot {\text{N}}_{\text{i}}\right]}$$ (6)

#### Biodiversity dynamics

Biodiversity dynamics within one community were modelled as:

$$\text{P}\left({\text{N}}_{\text{j}}+1|{\text{N}}_{\text{j}}\right)=\left[1-P\left(death\right)\right]\cdot \left[\left[1-m\right]\cdot P\left(reproduction\right)+m\cdot P\left(mainland\right)\right]$$ (7)

$$\text{P}\left({\text{N}}_{\text{j}}-1|{\text{N}}_{\text{j}}\right)=P\left(death\right)\cdot \left[\left[1-m\right]\cdot \left[1-P\left(reproduction\right)\right]+m\cdot \left[1-P\left(mainland\right)\right]\right]$$ (8)

with *P*(*mainland*) the relative abundance of species *j* in the mainland.

Note that Eqs 7 and 8 are not complements. Indeed, the abundance of a species can also stay constant with probability 1 − P(N_j_ + 1|N_j_) − P(N_j_ − 1|N_j_).

Substituting the different probabilities in Eqs 7 and 8 with those presented in Eqs 1–3, we can now specify biodiversity dynamics of one community that is experiencing biotic and abiotic stress as a set of two equations that give the probability of a species *j* to increase and decrease with one individual, respectively:

$$\text{P}\left({\text{N}}_{\text{j}}+1|{\text{N}}_{\text{j}}\right)=\left[1-\left[\frac{\text{P}\left[{\left(\text{c}>{\text{c}}_{\text{m}}\right)}_{\text{j}}\cup {\left({\left(f\times n\right)}_{\text{j}}>{\text{c}}_{\text{p}}\right)}_{\text{j}}\right]\cdot {\text{N}}_{\text{j}}}{\sum_{\text{i}=1}^{\text{n}}\left[\text{P}\left[{\left(\text{c}>{\text{c}}_{\text{m}}\right)}_{\text{i}}\cup {\left({\left(f\times n\right)}_{\text{i}}>{\text{c}}_{\text{p}}\right)}_{\text{i}}\right]\cdot {\text{N}}_{\text{i}}\right]}+\frac{{\text{N}}_{\text{j}}}{\sum_{\text{i}=1}^{\text{n}}{\text{N}}_{\text{i}}}-\frac{\text{P}\left[{\left(\text{c}>{\text{c}}_{\text{m}}\right)}_{\text{j}}\cup {\left({\left(f\times n\right)}_{\text{j}}>{\text{c}}_{\text{p}}\right)}_{\text{j}}\right]\cdot {\text{N}}_{\text{j}}}{\sum_{\text{i}=1}^{\text{n}}\left[\text{P}\left[{\left(\text{c}>{\text{c}}_{\text{m}}\right)}_{\text{i}}\cup {\left({\left(f\times n\right)}_{\text{i}}>{\text{c}}_{\text{p}}\right)}_{\text{i}}\right]\cdot {\text{N}}_{\text{i}}\right]}\cdot \frac{{\text{N}}_{\text{j}}}{\sum_{\text{i}=1}^{\text{n}}{\text{N}}_{\text{i}}}\right]\right]\cdot \left[\left(1-\text{m}\right)\cdot \left[\frac{\text{P}\left({\left({\left(f^{\text{T}}\;\text{x}\;n\right)}_{\text{j}}>{\text{c}}_{\text{f}}\right)}_{\text{j}}\cap {\left(\text{c}<{\text{c}}_{\text{r}}\right)}_{\text{j}}\right)\cdot {\text{N}}_{\text{j}}}{\sum_{\text{i}=1}^{\text{n}}\left[\text{P}\left({\left({\left(f^{\text{T}}\;\text{x}\;n\right)}_{\text{i}}>{\text{c}}_{\text{f}}\right)}_{\text{i}}\cap {\left(\text{c}<{\text{c}}_{\text{r}}\right)}_{\text{i}}\right)\cdot {\text{N}}_{\text{i}}\right]}\right]+\text{m}\cdot {\text{P}}_{\text{j}}\right]$$ (9)

and

$$\text{P}\left({\text{N}}_{\text{j}}-1|{\text{N}}_{\text{j}}\right)=\left[\frac{\text{P}\left[{\left(\text{c}>{\text{c}}_{\text{m}}\right)}_{\text{j}}\cup {\left({\left(f\times n\right)}_{\text{j}}>{\text{c}}_{\text{p}}\right)}_{\text{j}}\right]\cdot {\text{N}}_{\text{j}}}{\sum_{\text{i}=1}^{\text{n}}\left[\text{P}\left[{\left(\text{c}>{\text{c}}_{\text{m}}\right)}_{\text{i}}\cup {\left({\left(f\times n\right)}_{\text{i}}>{\text{c}}_{\text{p}}\right)}_{\text{i}}\right]\cdot {\text{N}}_{\text{i}}\right]}+\frac{{\text{N}}_{\text{j}}}{\sum_{\text{i}=1}^{\text{n}}{\text{N}}_{\text{i}}}-\frac{\text{P}\left[{\left(\text{c}>{\text{c}}_{\text{m}}\right)}_{\text{j}}\cup {\left({\left(f\times n\right)}_{\text{j}}>{\text{c}}_{\text{p}}\right)}_{\text{j}}\right]\cdot {\text{N}}_{\text{j}}}{\sum_{\text{i}=1}^{\text{n}}\left[\text{P}\left[{\left(\text{c}>{\text{c}}_{\text{m}}\right)}_{\text{i}}\cup {\left({\left(f\times n\right)}_{\text{i}}>{\text{c}}_{\text{p}}\right)}_{\text{i}}\right]\cdot {\text{N}}_{\text{i}}\right]}\cdot \frac{{\text{N}}_{\text{j}}}{\sum_{\text{i}=1}^{\text{n}}{\text{N}}_{\text{i}}}\right]\cdot \left[\left(1-\text{m}\right)\cdot \left[\frac{\text{P}\left({\left({\left(f^{\text{T}}\;\text{x}\;n\right)}_{\text{j}}<{\text{c}}_{\text{f}}\right)}_{\text{j}}\cap {\left(\text{c}>{\text{c}}_{\text{r}}\right)}_{\text{j}}\right)\cdot {\text{N}}_{\text{j}}}{\sum_{\text{i}=1}^{\text{n}}\left[\text{P}\left({\left({\left(f^{\text{T}}\;\text{x}\;n\right)}_{\text{i}}<{\text{c}}_{\text{f}}\right)}_{\text{i}}\cap {\left(\text{c}>{\text{c}}_{\text{r}}\right)}_{\text{i}}\right)\cdot {\text{N}}_{\text{i}}\right]}\right]+\text{m}\cdot {\left(1-\text{P}\right)}_{\text{j}}\right]$$ (10)

### Model simulations

We implemented Eqs 9 and 10 for one community of prey species and one community of predator species (four equations in total). Prey served as resource for predators but were themselves not resource limited (as in Eq 2). Predators were not predated. We ran this model in a full factorial design of three factors (type of abiotic stress, type of biotic stress and initial connectance) (Table 1). We considered three levels of abiotic stress: no abiotic stress on prey or predators, abiotic stress affecting prey reproduction only, or abiotic stress affecting predator reproduction only. We considered three levels of biotic stress: top-down control (predation pressure reduces prey survival), bottom-up control (resource limitation reduces predator reproduction), or both. We considered two levels of initial connectance: low (0.05 and high (0.20). We calculated connectance as L/(S_*prey*_ × S_*pred*_), where L is the number of links, S_*prey*_ is prey richness and S_*pred*_ is predator richness. Prey cannot eat predators or other prey and predators cannot eat other predators. The average number of links per species for initial conditions can, therefore, be calculated as 50×50×0.05 i.e. 125 (low) and 50×50×0.2 i.e. 500 (high). In literature, connectance is generally calculated for non-bipartite food webs with the formula L/S^2^, where L is the number of links and S is the total number of species [26,34–37]. The range of connectance used in our simulations (0.05–0.2), even after recalculation using the formula L/S^2^, corresponds to the ranges obtained in literature for non-bipartite food webs (0.026–0.315 [26], 0.061–0.32 [35], 0.026–0.122 [36] and 0.016–0.33 [38]).

**Table 1:** Model parameters in their initial state.

Description	Parameter	Level	Values
Total number of species (Constant)	n	Community	100 (50 predators; 50 prey)
Number of individuals: multiple of n (Variable)	N	Community	n×5
Level of abiotic stressor (Constant)	c	Community	0 or 100
Lower and upper limits of uniform distribution of immigration rate (Variable)	m_min_; m_max_	Individual	1E-01;1.5E-01 (low), 2E-01;3E-01 (high)
Lower and upper limits of critical reproduction threshold (Variable)	c_rmin_; c_rmax_	Individual	5E1;1.5E2
Lower and upper limits of food limitation and predation thresholds (c_f_ values constant and c_p_ values variable for top-down control; c_f_ values variable and c_p_ values constant for bottom-up control) [39]	c_fmin_; c_fmax_;	Individual	1E-10;1E-10; 10;40 (top-down control and predation pressure, no food limitation)
	c_pmin_; c_pmax_		10;40; 1E10;1E10 (bottom-up control and food limitation, no predation pressure)
Lower and upper limits of the slope of the stress response function (Constant) [42]	minslope; maxslope	Individual	3;3
Initial connectance (Initial connectance is constant; realised connectance is variable) [23,35,36,38]	Connectance	Community	Low (0.05) High (0.2)

Per combination of factor levels, Eqs 9 and 10 were numerically calculated and updated per time step for every prey species but only per four time steps for every predator species to simulate slower community dynamics for predators [39]. As such, the probabilities for abundance increase and decrease were obtained for every species by solving Eqs 9 and 10, respectively. Even though all prey species use the same implementations of Eqs 9 and 10, their tolerances to the stressors, their connections to predators, and their relative abundance in the mainland will be different. So, species-specific solutions will be obtained. The same holds for predators. Per time step (prey) or four timesteps (predators), one species was drawn per community as a weighted sample with the probability given by Eq 9. The abundances of these two species were increased with one. Next, one species was drawn per community as a weighted sample with the probability given by Eq 10. The abundances of these two species were decreased by one. This procedure assumes “zero sum dynamics” i.e. the number of individuals per community stays constant and at each time step, 1 individual per community is killed and replaced by a “new” individual. Because the model is dynamic, community composition and abundance of both prey and predators change over time. This implies that the levels of biotic stress also change over time, while abiotic stress is constant through time.

Per combination, the model was run (i.e. the probabilities were calculated) for 15000 time steps, using 5000 iterations. These 5000 iterations differed in the species-mean tolerances (Table 1). Initial species abundances (and therefore initial richness) were equal across all combinations and iterations. Food-web links were set at random with the initial connectance as the only constraint. Links between two species disappear when one of the species goes extinct locally but reappear when this species recolonizes the local community following an immigration event. No new links are created. For every combination and iteration we calculated the final prey and predator richness as well as final connectance. The model was coded in Python (2.7.10).

To assess the robustness of the simulations to the selected immigration probability *m*, we performed all simulations for two different values of *m* (Table 1). All figures were prepared using R [40]. Statistical tests were not used to interpret model simulation results as has been recommended recently [41].

## Results

**Fig 1:**
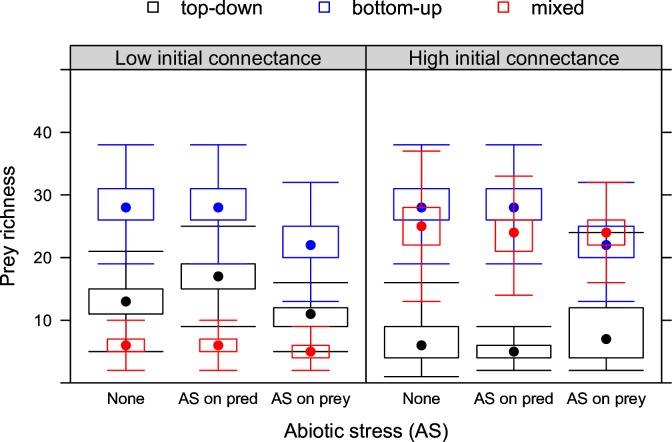
Prey richness under stress. Biotic stress (top-down, bottom-up or mixed control) and abiotic stress (none, AS on prey, AS on predators) at low and high initial connectance.

**Fig 2:**
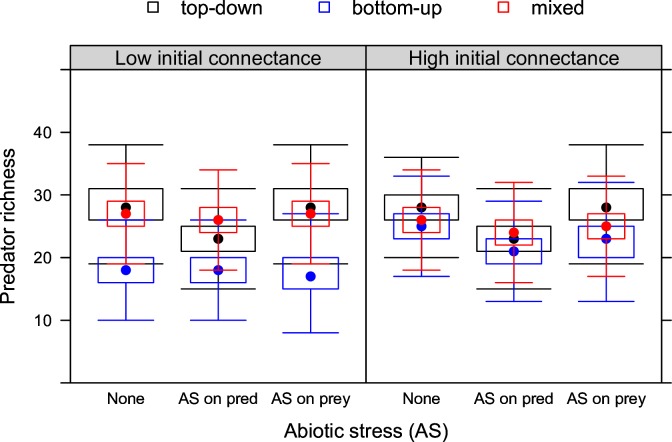
Predator richness under stress. Biotic stress (top-down, bottom-up or mixed control) and abiotic stress (none, AS on prey, AS on predators) at low and high initial connectance.

**Fig 3:**
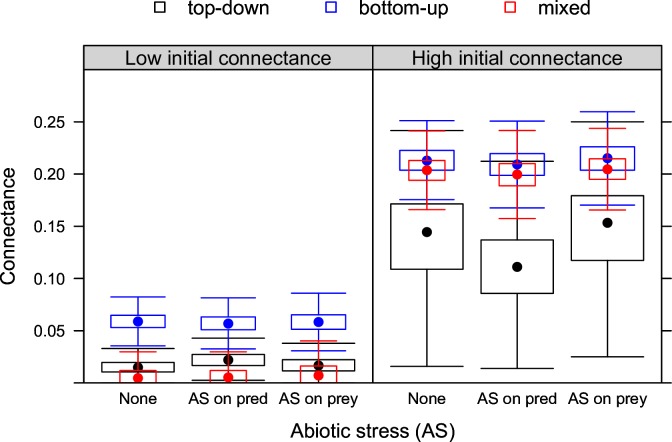
Connectance under stress. Biotic stress (top-down, bottom-up or mixed control) and abiotic stress (none, AS on prey, AS on predators) at low and high initial connectance.

**Fig 4:**
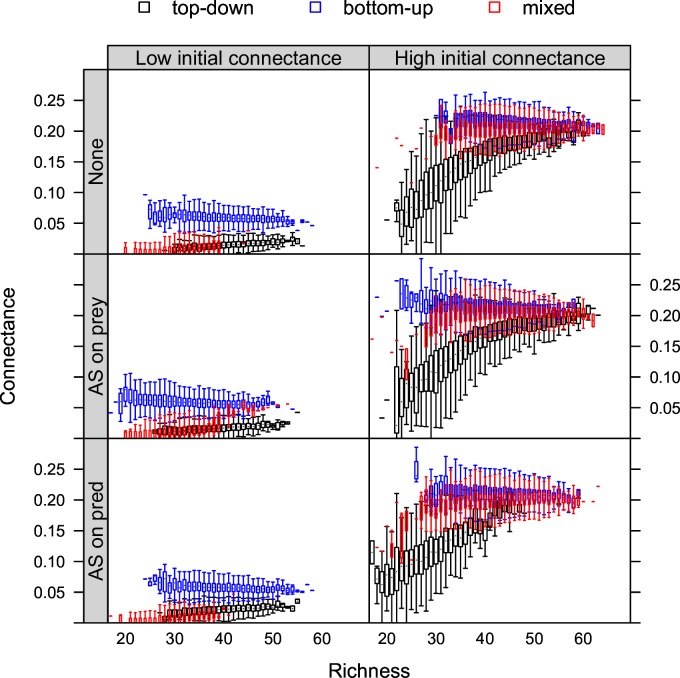
The relationship between connectance and total richness (prey and predators together) under stress. Biotic stress (top-down, bottom-up or mixed control) and abiotic stress (none, AS on prey, AS on predators) at low and high initial connectance.

Because results were qualitatively similar between lower (Figs 1–4) and higher immigration probabilities *m* (S1–S4 Figs), only the former are described and discussed below.

### Richness

#### Prey richness

In the absence of abiotic stress, prey richness under bottom-up control can be considered a reference (unstressed) situation, since prey also do not experience any biotic stress under bottom-up control. Thus, prey richness is only determined by dispersal limitation. Compared to this reference, top-down and mixed control decreased prey richness (Fig 1). Mixed control caused reductions of prey diversity that were more severe when initial connectance was low.

When abiotic stress affected prey, prey richness was reduced, but only in absence of biotic stress on prey (bottom-up control). When abiotic stress affected predators, prey richness increased under top-down control, but only at low initial connectance. At high initial connectance, no indirect effect on prey richness was observed.

#### Predator richness

In absence of abiotic stress, predator richness under top-down control can be considered an unstressed situation, since predators also do not experience any biotic stress under top-down control. Thus, bottom-up control reduced predator richness and did so most when initial connectance was low. Mixed control had no clear effect on predator richness (Fig 2).

A direct negative effect of abiotic stress on predator richness was found, but this effect was only pronounced in absence of biotic stress on predators (top-down control), regardless of initial connectance. When abiotic stress affected prey and bottom-up control prevailed, a small negative indirect effect on predator richness occurred but only at high initial connectance.

### Connectance

Whether or not abiotic stress was present, connectance was always lower under top-down than under bottom-up control (Fig 3). Connectance was lowest under mixed control at low initial connectance compared to top-down and bottom-up control but higher than that under top-down control at high initial connectance. Overall, abiotic stress did not markedly change connectance. Only when abiotic stress affected predators was connectance higher (lower) under top-down control when initial connectance was low (high).

In absence of abiotic stress, top-down control decreased the number of predators per prey (S5 Fig) while bottom-up control slightly increased the number of prey per predator. Mixed control decreased the number of predators per prey as well the number of prey per predator and both were very low at low initial connectance.

Both types of abiotic stress reduced the number of prey per predator under bottom-up control (S6 Fig). Under top-down control, abiotic stress affecting predators reduced the number of predators per prey (S7 Fig). Under mixed control, abiotic stress did not change the number of prey and predators per predator and prey, respectively (S6 and S7 Figs).

### Relationship between connectance and richness

The shape of the relationship between connectance and total richness (all species, including predators and prey) was strongly influenced by initial connectance and the type of food-web control, but was robust to the two types of abiotic stress considered here. At low initial connectance, irrespective of the presence or absence of abiotic stress, connectance remained constant under bottom-up control and showed a weak positive relationship under top-down and mixed controls with increasing total richness. At high initial connectance, we found a positive saturating relationship between connectance and richness under top-down and mixed control (Fig 4).

## Discussion

### Prey richness

Abiotic and biotic stress (top-down control) had direct negative effects on prey richness, which confirms empirical findings [43–45] and results from a recent meta-analysis [4], respectively. Effects of biotic stress on prey richness were more severe under high than under low initial connectance, because prey were by definition–on average–connected to more predators under high initial connectance. The effects of mixed control on prey richness illustrates that bottom-up control can aggravate or buffer negative effects of top-down control on prey richness, depending on whether initial connectance is low or high, respectively.

Food web interactions are known to cause indirect effects of abiotic stress on the density of non-target communities [17], but indirect effects on richness have been less well studied [4]. Empirical studies that have manipulated predator diversity do exist [26,46–48], but it is often difficult to manipulate diversity without manipulating density so that isolating effects of richness from density effects becomes difficult. In our theoretical study, we were able to only manipulate predator richness because the model postulates a constant size of the predator community. Our results therefore illustrate indirect effects on prey diversity (Figs 1 and 2) that are only due to abiotic stress affecting predator diversity and not density.

Connectance is considered a proxy for resistance of food-webs to indirect effects on density [5,26]. Food webs with low connectance are extremely sensitive and more prone to selective loss of highly connected nodes than food webs with high connectance [26]. Our results suggest that connectance also increases resistance against indirect effects on richness because we find that higher initial connectance increased resistance of prey diversity to indirect effects of abiotic stress on predators.

We did not find indirect effects on prey richness under mixed control (Fig 1). When abiotic stress affected predators, the subsequent increase in prey richness resulted in a higher number of prey species available to predators, which apparently resulted in a stabilizing feed-back mechanism. This shows that under mixed control, our framework predicts non-additive effects of biotic and abiotic stress in bipartite graphs.

### Predator richness

Abiotic and biotic stress (bottom-up control) decreased predator richness, which corresponds to empirical findings [49]. Effects of biotic stress was more pronounced at low than at high initial connectance (Fig 2) because predators have fewer feeding options at lower initial connectance and thus experience higher biotic stress. Empirical results [50] for bottom-up control on predator density suggest similar mechanisms. The effects of mixed control on predator richness illustrate that the negative effects of bottom-up control on predator richness are (partly) offset by feedback mechanisms from top-down control of fewer predator species on prey. This mechanism occurred regardless of initial connectance.

We did not find strong support for indirect effects of abiotic stress on predator richness (Fig 2). Under bottom-up control, abiotic stress on prey only slightly reduced predator richness and only when initial connectance was high. This can be explained by the fact that bottom-up control selects for predators feeding on multiple prey species. Because the probability to be connected to a tolerant prey (that can compensate for density loss of sensitive prey species) increases with the number of prey species in the diet, bottom-up control effectively reduces the probability for indirect effects.

### Connectance

Top-down control decreased connectance (Fig 3). This result is a logical consequence of selection of this type of biotic stress against highly connected prey species. This is illustrated by the negative effect of top down control on the number of links per predator species (S5 Fig). Bottom-up control, in contrast, increased connectance (Fig 3) by selecting against poorly connected predator species, as shown by the number of prey per predator (Fig). This result can have important implications for food-web stability [50–51] as the disappearance of highly or poorly connected species can cause significant changes in food web structure [5,26,52].

The negative effect of top-down control on the number of links per predator can also be interpreted as a higher degree of specialization emerging as the sole consequence of intense top-down control. Intense top-down interactions have been associated with a higher degree of specialization in the tropics [53]. This association has been explained by narrower niches in the tropics than at higher latitudes [54–55]. Our results indicate that intense top-down control could be the cause, rather than the result, of specialization.

Effects of mixed control on connectance reflected the effects on prey richness, suggesting that in the system we studied connectance was more determined by the number of prey species available than by the number of predators (Fig 3). At low initial connectance, mixed control shaped a prey community that was dominated by a low number of poorly connected species, while at high initial connectance this did not occur. This is evident from the distribution of predators per prey species (S5–S7 Figs).

We did not find any consistent effect of abiotic stress on connectance. This is a logical consequence from the absence of any correlation between the number of links and abiotic stress tolerance of species in our model. In real systems, such correlations can emerge as a result of body size difference, for example, where large bodied predators are more generalist [56] and at the same time are more resistant to certain types of abiotic stress such as desiccation [57].

### Relationship between connectance and richness

In absence of abiotic stress, the relationships between connectance and richness resulted from a richness gradient that was primarily caused by variability of the immigration rate because, by design, community-mean tolerance to biotic stress within a treatment was constant among iterations (Fig 4). Interestingly, this random richness gradient resulted in both negative (bottom-up) and positive richness-connectance relationships (other types of control). This result illustrates that, for the case of randomly composed communities, the often found negative relationship between richness and connectance [21–22] is not always supported, especially when top-down control prevails (Fig 4).

The effect of initial connectance on the richness-connectance relationship under top-down control can be understood from the effect of initial connectance on the richness gradient caused by random immigration. When initial connectance was high, there were more nonzero elements in the food web matrix, such that top-down control had a higher probability to remove prey species, which leads to a broader richness gradient (Fig 4). Food-web rewiring along environmental gradients has been often observed [58–59]. Our results show that such events may alter richness-connectance relationships by changing richness gradients.

### Outlook

Our model includes a minimal set of mechanisms only. Our implementation of abiotic stress only represents effects of chronic abiotic stress that persistently change reproduction, not of sudden events of acute stress (e.g. mass mortality). However, the model could be extended with a series of additional processes. First, the total community size could be made variable to account for changes in basal resource availability and space. This extension will be particularly important to account for control on prey and predator density. Second, the model assumes a bipartite graph. We only consider two trophic levels in our model and therefore, do not focus on higher-order interactions. Adding additional trophic levels would be interesting to study indirect effects in a multitrophic context [60–61]. In particular, we are aware that our model only represents cases where predator communities consist of species with similar life history traits, such that differences in mortality rates between species can be considered stochastic, even when driven by environmental processes not explicitly accounted for in the model. Thus, our model is unable to represent cases where predator species have different life histories and/or respond differently to environmental cues. The model can also be modified to explore how different implementations of abiotic stress can change the dynamics of the community by investigating various real life stressors that affect different species differently as well as focusing on incoming and outgoing degree distributions.

## Supporting information

S1 Fig Prey richness under stress with higher immigration probability *m*. Biotic stress (top-down, bottom-up or mixed control) and abiotic stress (none, AS on prey, AS on predators) at low and high initial connectance.
(PDF)

S2 Fig Predator richness under stress with higher immigration probability *m*. Biotic stress (top-down, bottom-up or mixed control) and abiotic stress (none, AS on prey, AS on predators) at low and high initial connectance.
(PDF)

S3 Fig Connectance under stress with higher immigration probability *m*. Biotic stress (top-down, bottom-up or mixed control) and abiotic stress (none, AS on prey, AS on predators) at low and high initial connectance.
(PDF)

S4 Fig The relationship between connectance and total richness (prey and predators together) under stress with higher immigration probability *m*. Biotic stress (top-down, bottom-up or mixed control) and abiotic stress (none, AS on prey, AS on predators) at low and high initial connectance.
(PDF)

S5 Fig Links per species in the absence of abiotic stress. L and H correspond to low (0.05) and high (0.2) connectance respectively. Solid lines denote initial distribution of links per species while dotted lines denote the realized links in the absence of abiotic stress.
(PDF)

S6 Fig Links per species under abiotic stress on prey. L and H correspond to low (0.05) and high (0.2) connectance respectively. Solid lines denote initial distribution of links per species while dotted lines denote the realized links in the presence of abiotic stress on prey.
(PDF)

S7 Fig Links per species under abiotic stress on predators. L and H correspond to low (0.05) and high (0.2) connectance respectively. Solid lines denote initial distribution of links per species while dotted lines denote the realized links in the presence of abiotic stress on predators.
(PDF)
