# Supplementary material for: The combined effects of biotic and abiotic stress on species richness and connectance
Source: PLoS One. 2017 Mar 1;12(3):e0172828. doi: 10.1371/journal.pone.0172828 (PMC5383007; doi:10.1371/journal.pone.0172828)
Supplement: S1 Fig — Biotic stress (top-down, bottom-up or mixed control) and abiotic stress (none, AS on prey, AS on predators) at low and high initial connectance. (PDF) [file pone.0172828.s001.pdf]

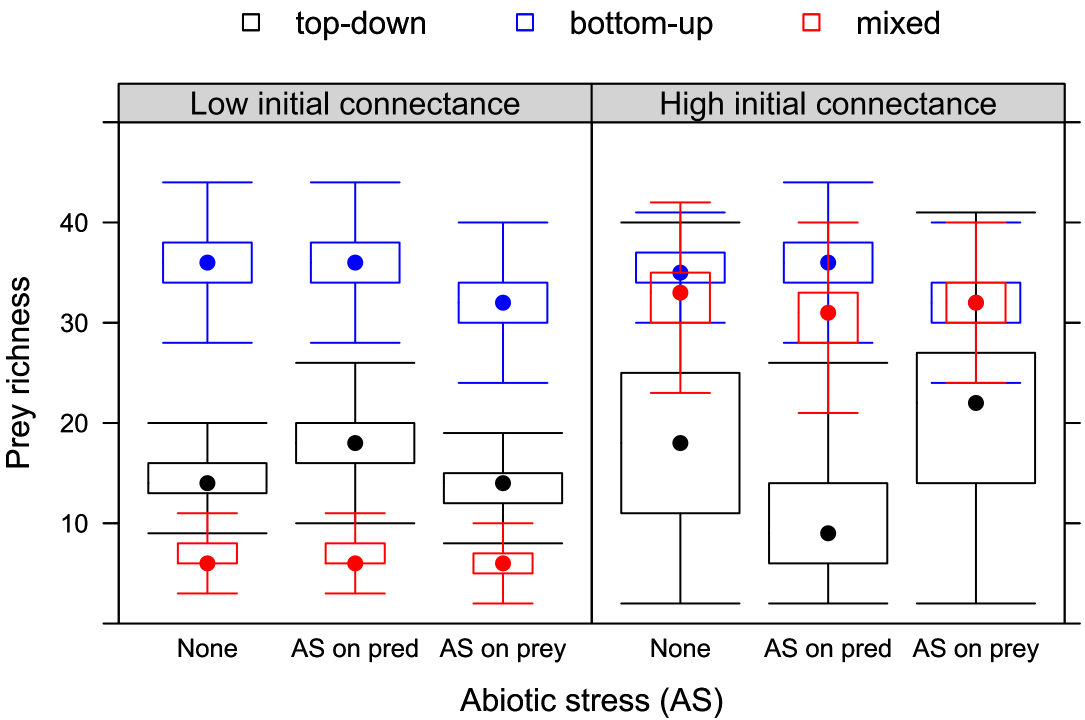
top-down
bottom-up
mixed
Low initial connectance
High initial connectance
Prey richness
40
30
20
10
None
AS on pred
AS on prey
None
AS on pred
AS on prey
Abiotic stress (AS)
