# Supplementary figures and images for: The combined effects of biotic and abiotic stress on species richness and connectance
Source: PLoS One. 2017 Mar 1;12(3):e0172828. doi: 10.1371/journal.pone.0172828 (PMC5383007; doi:10.1371/journal.pone.0172828)

□ top-down      □ bottom-up      □ mixed

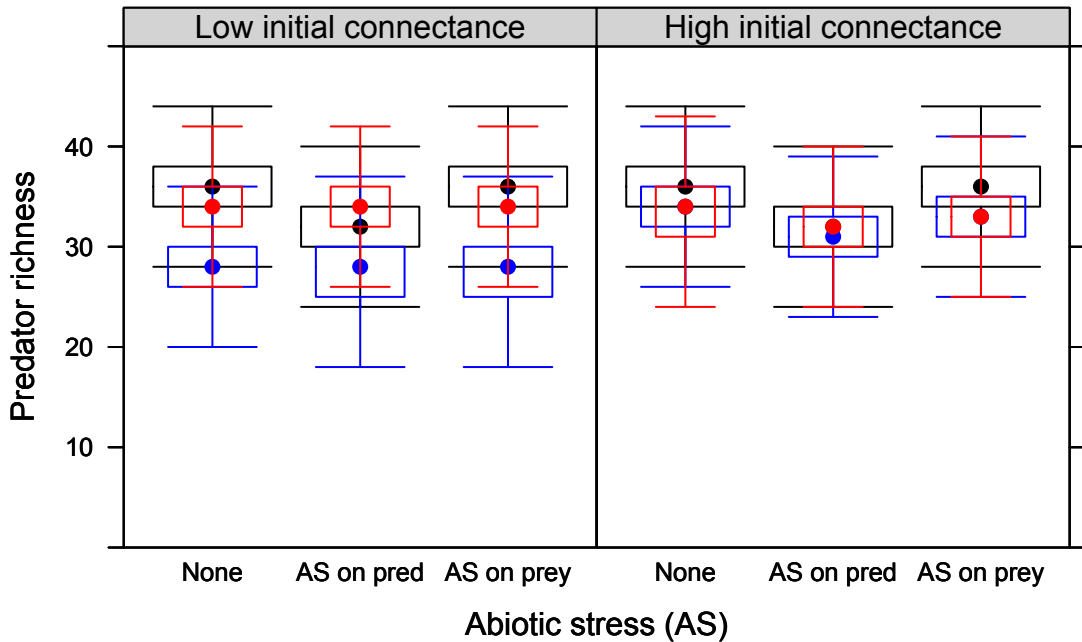

Supplement: S2 Fig — Biotic stress (top-down, bottom-up or mixed control) and abiotic stress (none, AS on prey, AS on predators) at low and high initial connectance. (PDF) [file pone.0172828.s002.pdf]

□ top-down      □ bottom-up      □ mixed

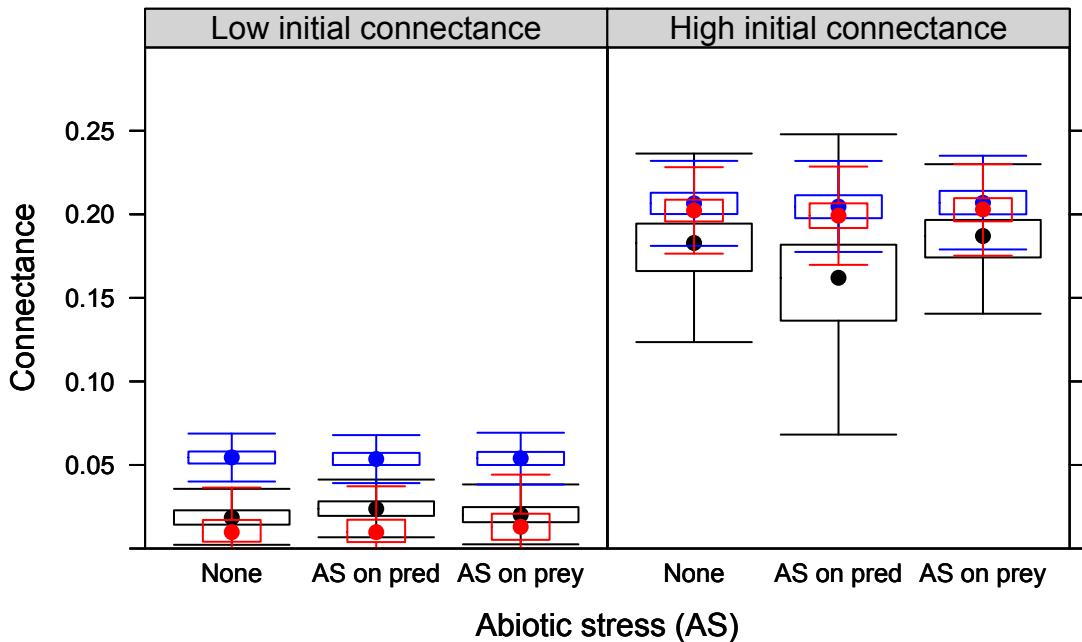

Supplement: S3 Fig — Biotic stress (top-down, bottom-up or mixed control) and abiotic stress (none, AS on prey, AS on predators) at low and high initial connectance. (PDF) [file pone.0172828.s003.pdf]

□ top-down      □ bottom-up      □ mixed

Connectance

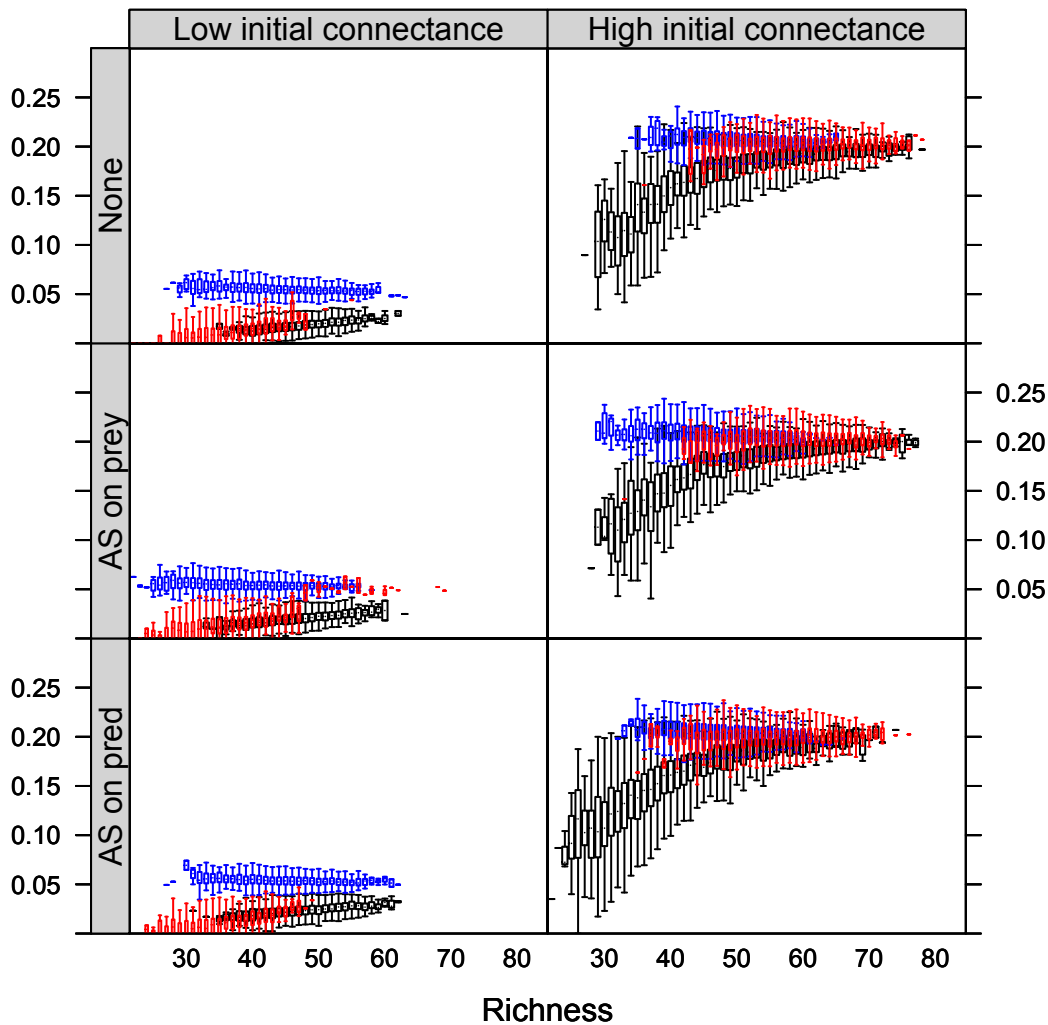

Supplement: S4 Fig — Biotic stress (top-down, bottom-up or mixed control) and abiotic stress (none, AS on prey, AS on predators) at low and high initial connectance. (PDF) [file pone.0172828.s004.pdf]
