# Supplementary material for: The combined effects of biotic and abiotic stress on species richness and connectance
Source: PLoS One. 2017 Mar 1;12(3):e0172828. doi: 10.1371/journal.pone.0172828 (PMC5383007; doi:10.1371/journal.pone.0172828)
Supplement: S6 Fig — L and H correspond to low (0.05) and high (0.2) connectance respectively. Solid lines denote initial distribution of links per species while dotted lines denote the realized links in the presence of abiotic stress on prey. (PDF) [file pone.0172828.s006.pdf]

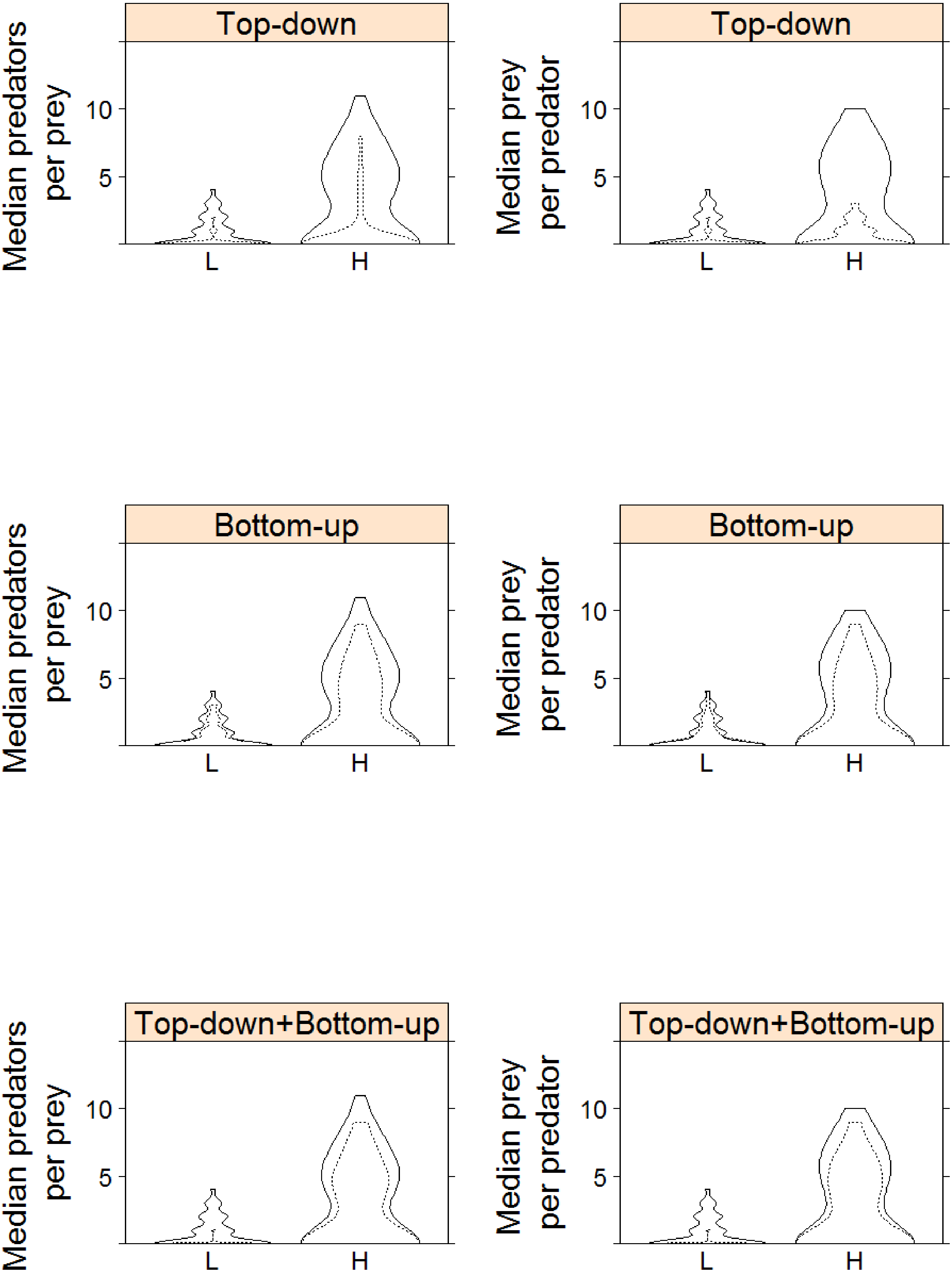

Top-down
Median predators per prey
10
5
L
H
Top-down
Median prey per predator
10
5
L
H
Bottom-up
Median predators per prey
10
5
L
H
Bottom-up
Median prey per predator
10
5
L
H
Top-down+Bottom-up
Median predators per prey
10
5
L
H
Top-down+Bottom-up
Median prey per predator
10
5
L
H
